# Effect of cold ischemic times and time after transplantation on regional myocardial motion after heart transplantation

**DOI:** 10.1186/1532-429X-15-S1-P99

**Published:** 2013-01-30

**Authors:** Daniela Föll, Michael Markl, Marius Menza, Asad Usman, Tobias Wengenmayer, Anna Lena Anjarwalla, Christoph Bode, James Carr, Bernd A Jung

**Affiliations:** 1 Cardiology and Angiology I, University Heart Center Freiburg, Freiburg, Germany; 2 Department of Radiology, Medical Physics, University Hospital Freiburg, Freiburg, Germany; 3 Department of Radiology, Feinberg School of Medicine, Northwestern University, Chicago, IL, USA; 4 Department of Biomedical Engineering, McCormick School of Engineering, Northwestern University, Chicago, IL, USA

## Background

Transplant rejection affects the course and survival after heart transplantation (HTx). As a non-invasive alternative to myocardial biopsy, which is the gold standard used for screening and for the diagnosis of rejection, regional left ventricular (LV) myocardial motion analysis has been suggested. But myocardial biopsy is invasive and its diagnostic value is restricted by high sample errors due to the patchy distribution of early rejection. Alterations of regional wall motion [1], especially in diastole, might be sensitive for the diagnosis. However, evaluation by echocardiography is limited in this context and LV motion after HTx differs from the motion of native hearts. We assessed the influence of cold ischemic time (CIT) and time after HTx on myocardial velocities in stable HTx patients.

## Methods

27 heart transplant recipients (50±13 years, 6 female) without signs or former episodes of rejections or relevant transplant vasculopathy (LVEF=63±5%) were examined using phase contrast MRI (Tissue Phase Mapping) to assess 3D-myocardial velocities. Systolic and diastolic peak radial and long-axis velocities were evaluated in each LV segment. These functional parameters were analyzed with respect to time after HTx and CIT.

## Results

**Figure 1:**
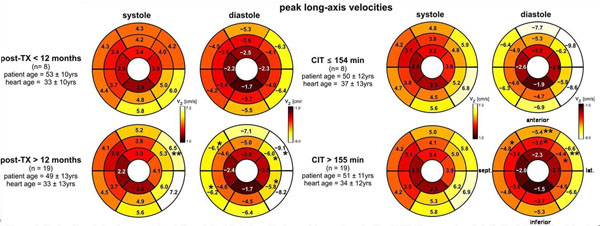
Regional analysis of segmental systolic and diastolic left ventricular peak long-axis (AHA 16-segment model). On the left side the data of the patients with shorter (post Tx<12 month, upper row) and longer time after heart transplantation (post Tx>12 month, lower row), on the right side of the data of the patients with shorter cold ischemic times (CIT≤154 minutes, upper row) compared to patients with longer cold ischemic times (CIT>155 minutes, lower row) are displayed. All data represent mean values over all patients after heart transplantation in the subgroup. ** and * indicate significant differences with p<0.01 resp P<0.05. Abbreviations: yrs: years; sept: septal; lat: lateral; Vz: long-asix velocities.

Shorter times after HTx (n=8 patients <12 month after HTx versus n=19 >12 month) resulted in an increase of systolic radial velocities (2 of 16 segments, p=0.01-0.04) and a reduction of diastolic long-axis velocities (5 of 16 segments, p=0.02-0.04). With longer CIT (>155 minutes) an increase of peak systolic radial velocities (p=0.03-0.04 for apico-septal and midventricular inferoseptal regions) and a reduction of diastolic long-axis velocities (5 of 16 segments, p=0.01-0.04) were demonstrated compared to those with shorter CIT (<155 min) (see figure 1, results of long-axis velocities).

## Conclusions

The transient suppression of long-axis velocities in the first months after HTx might be related to an early ischemia-reperfusion injury after cardiac surgery. The different behavior of long-axis and radial velocities with respect to time after HTx or CIT could be explained by the locations of the myocardial fibers underlying each motion component. Long-axis motion being mainly based on subendocardially located fibers might predispose this motion component for ischemic injury whereas circumferentially orientated fibers underlying radial motion are predominantly situated in the midmyocardium and might therefore have more functional reserves [2].

Time after HTx and CIT influence segmental systolic and diastolic velocities in stable patients after heart transplantation. These findings have to be considered if myocardial velocities are used as diagnostic tools in transplant rejection.

## Funding

NMH Excellence in Academic Medicine (EAM) Program "Advanced Cardiovascular MRI Research Center", Deutsche Forschungsgemeinschaft (DFG): Grant # FO 507/2-1, FO 507/3-1
